# Molecular Aggregation Strategy for Pore Generation in SiOC Ceramics Induced by the Conjugation Force of Phenyl

**DOI:** 10.3390/polym15122676

**Published:** 2023-06-14

**Authors:** Gang Yi, Yuxi Yu

**Affiliations:** Fujian Key Laboratory of Advanced Materials, Department of Materials Science and Engineering, College of Materials, Xiamen University, Xiamen 361005, China; yigang009@163.com

**Keywords:** SiOC ceramics, molecular phase separation, macroporous, tailorable microstructure, phenyl-substituted cyclosiloxane

## Abstract

Porous silicon oxycarbide (SiOC) ceramics with tailorable microstructure and porosity were fabricated using phenyl-substituted cyclosiloxane (C-Ph) as a molecular-scale porogen are analyzed in this study. A gelated precursor was synthesized via the hydrosilylation of hydrogenated and vinyl-functionalized cyclosiloxanes (CSOs), followed by pyrolysis at 800–1400 °C in flowing N_2_ gas. Tailored morphologies, such as closed-pore and particle-packing structures, with porosities in the range 20.2–68.2% were achieved by utilizing the high boiling point of C-Ph and the molecular aggregation in the precursor gel induced by the conjugation force of phenyl. Moreover, some of the C-Ph participated in pyrolysis as a carbon source, which was confirmed by the carbon content and thermogravimetric analysis (TGA) data. This was further confirmed by the presence of graphite crystals derived from C-Ph, as determined by high-resolution transmission electron microscopy (HRTEM). In addition, the proportion of C-Ph involved in the ceramic process and its mechanism were investigated. The molecular aggregation strategy for phase separation was demonstrated to be facile and efficient, which may promote further research on porous materials. Moreover, the obtained low thermal conductivity of 27.4 mW m^−1^ K^−1^ may contribute to the development of thermal insulation materials.

## 1. Introduction

Silicon oxycarbide (SiOC) ceramics are carbon-containing silicates consisting of glass networks in which oxygen and carbon share bonds with silicon. The carbon substitution of oxygen in silicate glass networks was demonstrated to induce considerable changes in network connectivity and, consequently, significant improvements in silicate glass network properties [1]. SiOC ceramics are considered “all-rounder” materials owing to their wide range of advanced functional and structural properties [2]. For example, SiOC ceramics exhibit Young’s moduli [3,4], hardness values [5,6], glass transition temperatures [7], and crystallization temperatures [8,9] that are superior to those of vitreous silica.

Porous SiOC ceramics have attracted increasing attention because of their tailorable permeability, low density, high thermal/chemical stability, and low thermal conductivity. They are considered potential candidates for various applications, such as thermal insulation at high temperatures [10], catalyst supports [11], hot gas separation [12], molten metal filtration [13], thermal transducers [14], high-temperature burners [15], and biomedical materials [16]. The porosity and morphology of ceramics strongly affect their application. For example, closed-pore ceramics, which have an ultralow heat transfer efficiency, are applied in thermal insulation materials, missile warheads, and aerospace planes [17]. Conversely, bicontinuous ceramics are suitable for advanced transportation applications owing to their unique permeability [18].

Liquid- and/or solid-soluble polymers have proven to be facile templates for the fabrication of porous SiOC ceramics; this method is defined as a template-assisted technique [19,20,21]. In particular, nontoxic and inexpensive methyl-terminated polysiloxane (PDMS) was mixed into sol-gel precursors to prepare tunable porous ceramics, owing to its decomposition into cyclic and linear dimethylsiloxane and complete release from the matrix by pyrolysis at intermediate temperatures (above 400 °C). Compared to the hard-templating techniques using SBA-15, CMK, and KIT-6 [22,23], PDMS avoids the introduction of unnecessary elements or impurities into the targeted porous materials. Hence, considering the advantages of PDMS and the abundance of silicon compounds, this study aims to explore other chemicals as porogens and to develop silicon chemistry in the field of porous materials.

Phenyl-substituted cyclosiloxane (C-Ph), a commercially available cyclosiloxane, has a high boiling point (above 400 °C) and is miscible with common polysiloxane precursors. At the molecular level, C-Ph has many advantages over polymers as a porogen in polymer-derived ceramics (PDCs). Most importantly, C-Ph is relatively inexpensive compared to polymers. Furthermore, C-Ph evaporates at lower temperatures, thereby minimizing the degradation of the polymer or ceramic matrix. Moreover, the dispersibility of small molecules in polymer matrices is usually better than that of polymers. Thus, C-Ph is suitable for the fabrication of various PDCs [24]. The porogens at the molecular level may flexibly control the morphology and composition of the porous ceramics. By varying the type, amount, and distribution of the porogens, the pore size, shape, volume fraction, and connectivity of the PDCs can be tuned. This can affect their performance in various applications, such as catalysis, gas separation, energy storage, and sensing. However, to the best of our knowledge, no reports have been published on the use of molecular-scale porogens in PDCs. Based on a reported study on porous SiOC materials using hydrogenated and vinyl-functionalized cyclosiloxanes (CSO) [25], it was hypothesized that C-Ph may be a novel molecular-scale porogen for fabricating porous materials.

In this study, porous SiOC ceramics with controllable morphologies and porosities are fabricated. C-Ph is used as a molecular-scale porogen because of its high solubility in CSO, which facilitates the uniform dispersion of C-Ph in the precursor solution. In addition, a strong conjugation between the phenyl structures facilitates microphase separation during gelation, which is beneficial for pore formation after pyrolysis. Residual porogens are analyzed by measuring the carbon contents of the ceramics. The mechanism of the molecular-scale porogen is studied. Additionally, samples with an appropriate pore size distribution are qualified for thermal insulation applications.

## 2. Materials and Methods

### 2.1. Materials

Tetramethylcyclotetrasiloxane (D_4_H; 98%), 1,3,5,7-tetravinyl-1,3,5,7-tetramethylcyclotetrasiloxane (D_4_Vi; 98%), and C-Ph (20–30% of cyclotrisiloxane, 55–65% of cyclotetrasiloxane, and 10–20% of cyclopentasiloxane) were purchased from Shandong Dongyue Co., Ltd. (Zibo, China). Karstedt Catalyst (5000 ppm) was purchased from Shin-Etsu Co., Ltd. (Tokyo, Japan).

### 2.2. Characterization

The pore size distributions were measured using AutoPore V 9620 mercury injection apparatus (Micromeritics, Norcross, GA, USA). High-resolution transmission electron microscopy (HRTEM) and selected area electron diffraction (SAED) were performed on a JEM-F200 transmission electron microscope (JEOL, Tokyo, Japan). Fourier-transform infrared spectroscopy (FTIR) spectra were collected using a Nicolet 5700 FTIR spectrometer (Thermo Electron Scientific Instruments Corp., Waltham, MA, USA). X-ray photoelectron spectroscopy (XPS) was performed using an ESCA Lab 220i-XL electron spectrometer (VG Scientific, London, UK). Scanning electron microscopy (SEM) images of the ceramics were obtained using a MERLIN compact scanning electron microscope (Zeiss, Oberkochen, Germany). X-ray diffraction (XRD) measurements were performed using an Ultima IV diffractometer (Rigaku, Tokyo, Japan). Thermogravimetric analysis (TGA) was performed using an STA 449F3 instrument (Netzsch, Germany) under argon flow in the temperature range from room temperature (rt, ~25 °C) to 1000 °C at a heating rate of 5 °C/min. Thermal conductivity data were collected using a TPS2200 instrument (Hot Disk, Gothenburg, Sweden). The linear shrinkages and the densities of the ceramics were calculated based on their dimensions. The carbon contents of the ceramics were measured using a CS-800 instrument (Eltra Gmbh, Neuss, Germany). ^29^Si solid-state nuclear magnetic resonance (^29^Si NMR) spectra of the SiOC ceramics were measured with an NMR spectrometer (Bruker AVANCE III HD 400 MHz) 79.48 MHz.

### 2.3. General Procedures

Mixtures of D_4_H (3.0 g, 0.0125 mol), D_4_Vi (4.3 g, 0.0125 mol), C-Ph (appropriate mass ratio, 0–29.2 g), and the Karstedt catalyst (50.0 ppm) were prepared. The homogeneous mixtures were poured into cylindrical molds, heated from 30 to 100 °C at a rate of 5 °C/15 min, and held for 3 h to form crosslinked siloxane gels. Subsequently, the siloxane gels were pyrolyzed in a tube furnace under an N_2_ atmosphere to fabricate the SiOC ceramics.

The typical heating process was as follows: the pyrolysis temperature was increased from rt to the maximum temperature at a rate of 5 °C/min. In this study, pyrolysis was performed at maximum temperatures of 800, 1000, 1200, and 1400 °C. After 1 h at the set maximum temperature, the tube furnace was cooled to rt at 2 °C/min.

## 3. Results and Discussion

**Figure 1:**
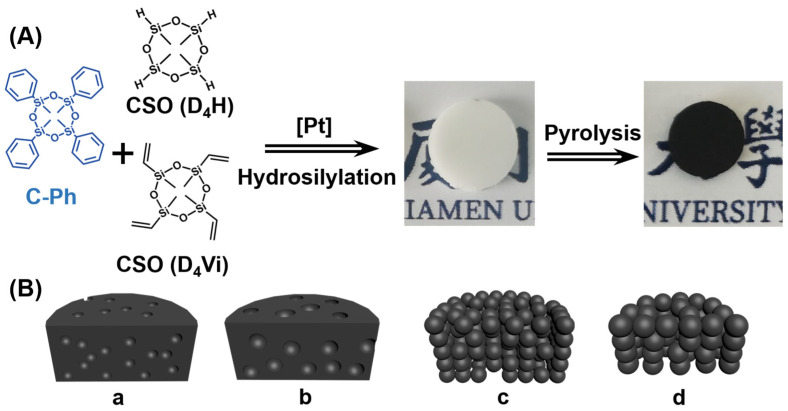
(**A**) Schematic for fabricating porous SiOC ceramics using C-Ph as the porogen. (**B**) SiOC ceramics pyrolyzed from precursors with different C-Ph contents, in increasing order from a to d.

As illustrated in Figure 1A, the ceramic precursor gels were synthesized via the platinum-catalyzed hydrosilylation of CSO. During gelation, C-Ph was homogeneously dispersed in CSO. The resulting gels became opaque upon hydrosilylation. Subsequently, pyrolysis was performed to fabricate SiOC ceramics with different C-Ph contents. Finally, ceramics with tailorable microstructures and porosities were obtained owing to the decomposition and elimination of C-Ph.

The mechanism by which the porogen adjusts the ceramic microstructure is illustrated in Figure 1B. The C-Ph porogen controlled the precursor gel morphology in three stages. During the first stage, the C-Ph content was less than 50 wt%, and CSO was in the continuous phase (insets a and b in Figure 1). When CSO solidified into the precursor gel, microphase separation occurred, and C-Ph aggregates formed in the gel framework. Subsequently, closed-pore structures formed by the volatilization and diffusion of C-Ph through the CSO gel framework during pyrolysis. The volume of the C-Ph aggregates increased with the increasing C-Ph content. During the second stage, the C-Ph content reached 50 wt%, forming a bicontinuous structure in the CSO/C-Ph solution. The reduced CSO content and increased C-Ph conjugation force prevented the CSO gel from compressing C-Ph into large droplets. Small CSO gel particles formed, inducing a particle-packing structure after pyrolysis (inset c of Figure 1). During the third stage (inset d in Figure 1), the C-Ph content continued to increase. When phase separation occurred because of CSO gel formation, the strong repulsion of the C-Ph conjugate force extruded the CSO phase to form fewer, larger particles. Under continuous heating, these larger particles gradually connect to form a particle network induced by molecular thermal motion.

**Figure 2:**
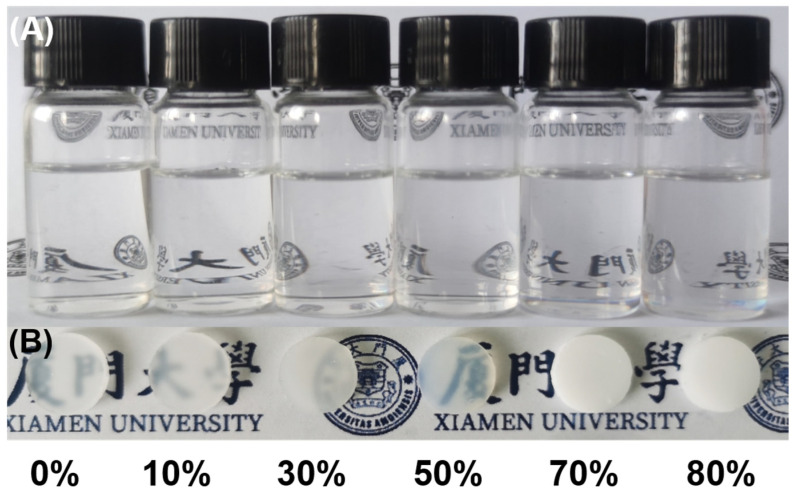
Photographs of the (**A**) C-Ph/CSO mixture (under gel preparation temperature, 100 °C) and (**B**) the corresponding precursor gels with different C-Ph contents.

To study the evolution of C-Ph as a molecular-scale porogen, different amounts of C-Ph were added to CSO. As shown in Figure 2A, the initial liquid mixtures are transparent, confirming the homogeneous dispersion of C-Ph in the mixtures under gel preparation temperature (100 °C). After hydrosilylation, the mixtures became opaque, and the crosslinked cyclosiloxanes were gradually whitened with increasing C-Ph content up to 80 wt% (Figure 2B). This may have been because hydrosilylation leads to the formation of crosslinked CSO gel frameworks, inducing phase separation between C-Ph and the CSO gel, which gradually intensifies as the C-Ph aggregates accumulate. Notably, the transparency of the precursor gel with 50 wt% C-Ph (denoted as precursor-50) was greater than that of precursor-30. This indicates that the degree of phase separation in the precursor gel decreased when the C-Ph content was increased from 30 wt% to 50 wt%.

**Figure 3:**
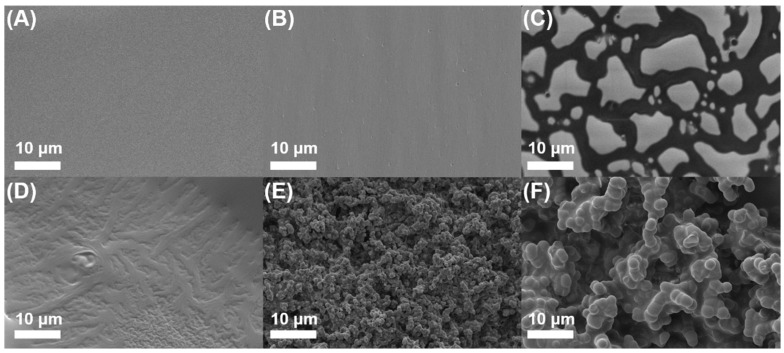
SEM images of the fractural cross-sections of the precursor gels with different C-Ph contents. (**A**–**F**) represent 0, 10, 30, 50, 70, and 80 wt%, respectively.

Furthermore, to evaluate C-Ph phase separation during gelation, SEM images of the precursor gels with different C-Ph contents are shown in Figure 3, and the corresponding magnification image of Figure 3D–F is presented in Figure S1. For precursor-0 (Figure 3A), a smooth surface was observed, whereas small pits appeared in precursor-10 (Figure 3B). For precursor-30 (Figure 3C), protrusions were observed, which may be due to C-Ph embedded in the precursor gel that protrudes from the surface under the negative pressure of SEM. These phenomena indicate that the degree of phase separation of the precursors gradually increases with increasing C-Ph content from 10 wt% to 30 wt%, and consequently, the precursor became increasingly opaque. For precursor-50 (Figure 3D and Figure S1A), only lines were observed, at which point the CSO/C-Ph bicontinuous structure appeared instead of droplet embedding. This may be due to the balance between the extrusion force of the CSO gel and the conjugation force of C-Ph, which supports the higher degree of phase separation in precursor-50 than that in precursor-30. A particle-packing structure was observed for precursor-70 (Figure 3E and Figure S1B), whereas larger particles were observed for precursor-80 (Figure 3F and Figure S1C). The interconnected particles significantly increased the degree of phase separation; thus, the precursor gels became completely opaque. The phase separation of the C-Ph aggregates and gel frameworks may induce various porous morphologies of the ceramic during pyrolysis.

**Figure 4:**
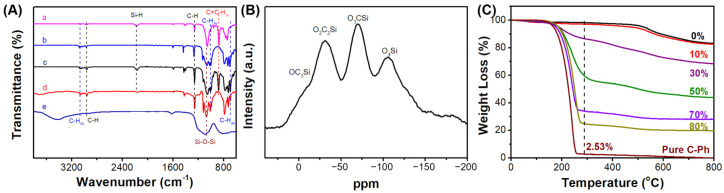
(**A**) FTIR spectra of (a) CSO, (b) C-Ph, (c) the liquid mixture of CSO and C-Ph, (d) crosslinking gels of CSO and C-Ph, and (e) ceramics using 70 wt% C-Ph as an example. (**B**) ^29^Si NMR spectra for S70 (the ceramic pyrolyzed using the CSO/C-Ph precursor with 70 wt% C-Ph content) pyrolyzed at 800 °C. (**C**) TGA curves of the precursor gels with different C-Ph contents.

In addition, FTIR was performed to confirm the C-Ph dispersion during the gelation and ceramic processes (Figure 4A). When the C-Ph content was 70 wt%, the absorbance peaks of the Si-H and C=C-vi groups were observed at 2170 and 891 cm^−1^, respectively, and were weakened after hydrosilylation. In comparison, those of the phenyl group of C-Ph were maintained at 697, 1016, and 3070 cm^−1^ during the gel transition process. These results indicate that C-Ph aggregation in the precursor gels is a prerequisite for the formation of the final porous SiOC ceramics. The complete absence of C–H bonds (1257 and 2954 cm^−1^) in the ceramics indicates that these bonds are cleaved during the ceramic process. The ^29^Si NMR spectrum of S70 pyrolyzed at 800 °C is illustrated in Figure 4B. Four types of silicon shifts were observed, namely, OSiC3 (~−10 ppm), O_2_SiC2 (~−30 ppm), O_3_SiC (~−70 ppm), and O_4_Si (~−110 ppm), which are similar to those reported elsewhere [26]. This indicates that SiOC structures were formed at 800 °C. The SiOC ceramics fabricated at 800 °C were characterized by energy-dispersive X-ray spectroscopy (EDX) mapping. As shown in Figure S2, Si, O, and C were uniformly distributed in the pink region, further confirming the successful synthesis of the SiOC ceramics at 800 °C.

To understand the thermal behavior during the polymer-to-ceramic transformation, ceramic precursor gels with different C-Ph contents were characterized by TGA, as shown in Figure 4C. Notably, the ceramic yield of S10 was comparable to that of S0, and pure C-Ph exhibited no residue at 800 °C. This phenomenon indicates that less C-Ph evaporation occurred during the pyrolysis of S10 and S20, which may be owing to the protective effect induced by the higher degree of CSO crosslinking [20]. The ceramic yield, calculated yield of the CSO phase, and the residual C-Ph phase contents are listed in Table 1. The residual C-Ph phase was detected in all the ceramics and was the lowest in S50. This may be owing to the insignificant phase separation in S50, which increases the volatility of C-Ph during pyrolysis.

**Figure 5:**
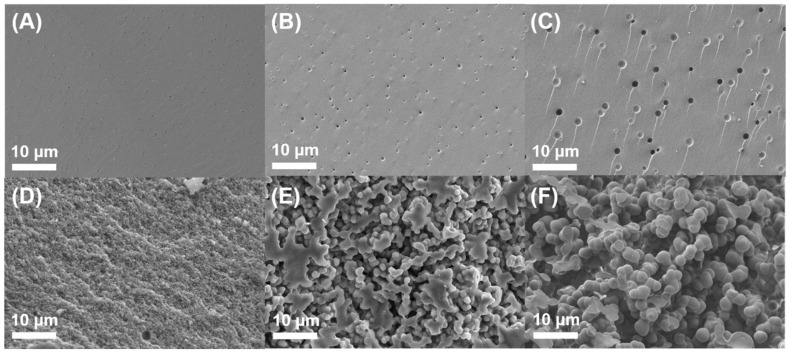
SEM images of the fractural cross-sections of SiOC ceramics S0 (**A**), S10 (**B**), S30 (**C**), S50 (**D**), S70 (**E**), and S80 (**F**) pyrolyzed at 800 °C.

The microstructures of S0–S80 are shown in Figure 5 (the corresponding magnification image was presented in Figure S3) and were similar to those of the precursor gels. Initially, fine pits were observed for S0, possibly caused by unreacted CSO or gas from cracking. The pits gradually increased with the addition of C-Ph up to 30 wt% (Figure 5A–C and Figure S3A–C). Notably, the yield of S10 was similar to that of S0; however, the size and number of pores of S10 were considerably greater than those of S0, which may be owing to the volatilization and diffusion of C-Ph to the CSO phase. When the C-Ph content was increased to 50 wt% (Figure 5D and Figure S3D), an agglomerated nanoparticle structure was obtained owing to the bicontinuous structure of the precursor gel. Moreover, when the C–Ph content was increased to 70 wt% and 80 wt%, particle-packing structures corresponding to the gel morphology were obtained (Figure 5E,F and Figure S3E,F).

**Figure 6:**
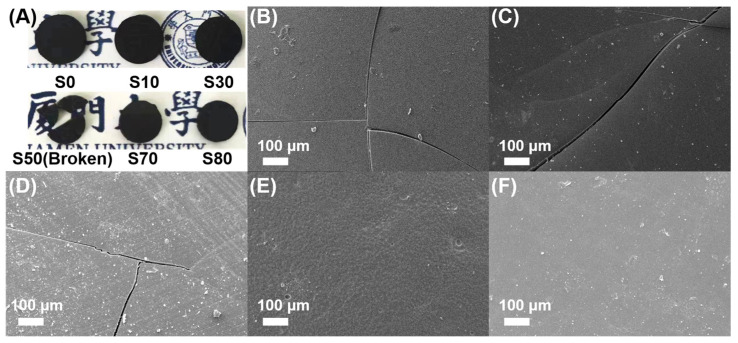
(**A**) Photographs of S0 to S80. (**B**–**F**) SEM images of the surfaces of S0 (**B**), S10 (**C**), S30 (**D**), S70 (**E**), and S80 (**F**).

Photographs of S0–S80 are shown in Figure 6A, where the SiOC ceramics appear as black solids. Notably, S50 exhibited structural damage, which was probably owing to its small particles that are prone to structural degradation upon pyrolysis; therefore, a further characterization of this sample will be discussed hereafter. SEM images of the surface morphologies of the SiOC ceramics are shown in Figure 6B–F, and the corresponding magnification image is presented in Figure S4. Microcracks were detected in S0–S30 (Figure 6B–D and Figure S4A–D), which is reasonable because SiOC ceramics formed at a rapid heating rate (5 °C/min) are prone to microcrack formation [27]. However, no cracks were detected when the C-Ph content was 70 wt% and 80 wt% (Figure 6E,F and Figure S4E,F). This phenomenon may be attributed to the fact that, when the concentration of C-Ph is low, the cross-linking density is high. The internal gas generated as well as vaporized C-Ph cause excessive internal pressure, which is difficult to discharge. However, under the same heating rate, increasing the proportion of C-Ph results in a lower cross-linking density. The morphology of particle stacking causes vaporized C-Ph and generated gas to be easily released, thereby preventing microcracks caused by imbalances in internal and external pressures.

**Figure 7:**
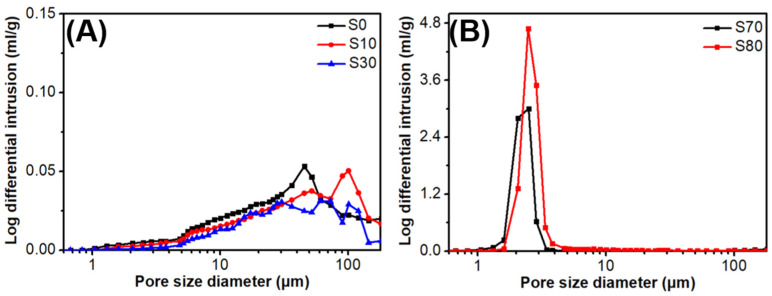
Pore size distributions of (**A**) S0, S10, and S30, and (**B**) S70 and S80.

To evaluate the porosity of the SiOC ceramics, the pore size distributions of S0, S10, S30, S70, and S80 fabricated at 800 °C are shown in Figure 7. For S0 to S30 (Figure 7A), the pore diameters were in the range 1–100 μm and exhibited low log differential intrusion values, which may be owing to microcrack formations in these ceramics. Notably, almost no intrusion occurred when the pore size was smaller than 1 μm, which confirms that the small holes observed by SEM correspond to closed pores (Figure 5B,C). For S70 and S80 (Figure 7B), a strong peak was observed at 2–3 μm, which corresponds to gaps between particles. The log differential intrusion and pore diameter of S80 were greater than those of S70, which indicates that the pore size and porosity of ceramics can be controlled by varying the C-Ph content. No peaks were observed between 10 and 100 μm, which further confirms the absence of cracks, as observed in the SEM images (Figure 6E,F).

Table 2 lists the average pore diameters, open porosities, bulk densities, porosities, carbon contents, and thermal conductivities of the ceramics (S0–S80). Notably, S50 was not analyzed because it was prone to degradation. For S0–S30, the pore diameters were in the range 8000–22,000 nm, which may be caused by microcracks. However, the open porosity gradually decreased from S0 to S30, which may be because C-Ph doping reduces the degree of crosslinking and the number of microcracks [28]. The open porosities (microcracks) of S0–S30 were significantly lower than their corresponding porosities, indicating that the small pores caused by C-Ph existed as closed pores in S0–S30. Thus, it was concluded that C-Ph was volatilized and diffused to the CSO phase, forming pores during pyrolysis. During diffusion, some of the C-Ph was captured by the CSO phase, which increased the carbon content of the ceramic. This is supported by the obtained carbon contents of the ceramics. Moreover, the carbon content of S0 was considerably lower than those of the other CSO/C-Ph-derived ceramics. Notably, the carbon content of S50 was lower than that of the other CSO/C-Ph-derived ceramics, which is consistent with the calculated residual C-Ph content results (Table 1). The thermal conductivities of S0–S80 decreased linearly with the increasing C-Ph content, and the lowest value (S80) was 27.4 mW m^−1^ K^−1^. The low thermal conductivity and high porosity of the material determines its suitability for porous thermal insulation materials.

**Figure 8:**
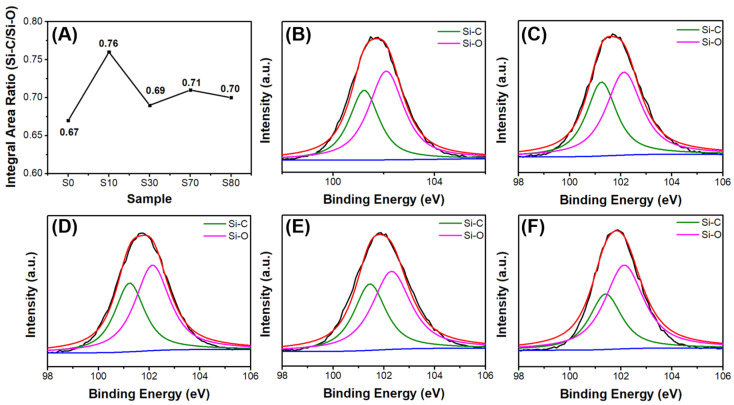
(**A**) XPS integral area ratio of Si-C/Si-O and (**B**–**F**) XPS spectra of Si for S0 (**B**), S10 (**C**), S30 (**D**), S70 (**E**), and S80 (**F**).

The XPS integral area ratios of Si-C/Si-O for S0–S80 are shown in Figure 8A. The Si-C/Si-O ratio of S0 was the lowest, whereas that of S10 was the highest, and those of the other ceramics remained relatively unchanged. This trend corresponds to the carbon contents of the ceramics. In addition, the compositions and functional groups of the SiOC ceramics were evaluated using XPS (Figure 8B–F), and the XPS peak fitting results of the narrow scan spectra of Si are presented in Table S1. The peaks at ~281 and ~282 eV were assigned to Si-C and Si-O, respectively.

**Figure 9:**
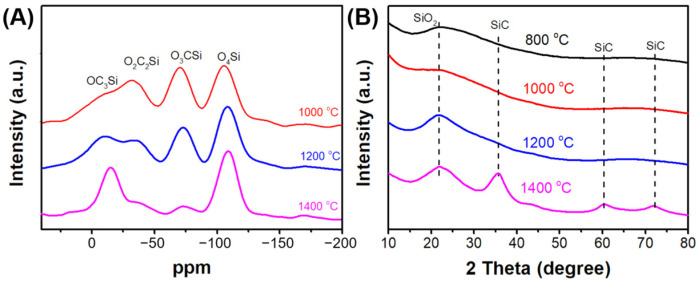
(**A**) The ^29^Si NMR and (**B**) XRD spectra for S70 pyrolyzed at different temperatures.

XRD and ^29^Si NMR were used to further characterize the ceramics. As the pyrolysis temperature increased, the ^29^Si NMR spectra (Figure 9A) exhibited an increase in the intensities of the OSiC_3_ and O_4_Si units, which was due to the carbothermal reduction in the silica matrix at high temperatures. The XRD results (Figure 9B) demonstrate that the SiOC ceramics consist of SiC and amorphous SiO_2_. The peaks located at 36.5°, 61.6°, and 71.7° correspond to the SiC phase, whereas that at 22.6° corresponds to amorphous SiO_2_. This result is owing to the increase in the degree of crystallization of SiC and SiO_2_ with increasing temperature.

**Figure 10:**
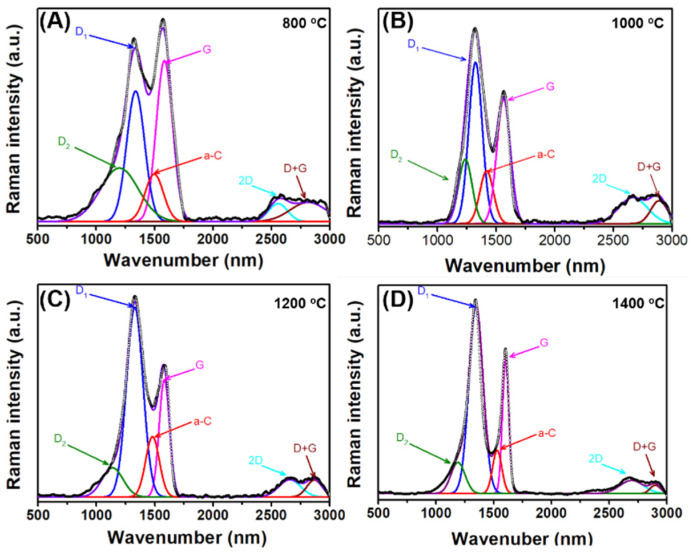
Raman spectra of S70 pyrolyzed at different temperatures, ((**A**–**D**) represent 800 to 1400 °C, respectively).

Subsequently, Raman spectroscopy was performed to analyze the structural defects of the ceramics fabricated at 800–1400 °C. As presented in Figure 10A–D and Table S2, the ID_1_/IG ratio was directly proportional to the fabrication temperature, which confirms that high carbonization temperatures induce defects in ceramics. Comparatively, the ID_2_/IG ratio decreased as the temperature was increased from 800 to 1000 °C, but remained stable when the temperature was increased to 1400 °C. This phenomenon may be due to graphitization occurring at temperatures of 800–1000 °C, which remained relatively stable at 1000–1400 °C. Moreover, the oxygen functional group content (Ia-C/IG) increased with increasing the temperature up to 1200 °C, which may be due to oxidation caused by residual oxygen in the ceramics, and subsequently decreased at 1400 °C owing to carbothermic reduction.

**Figure 11:**
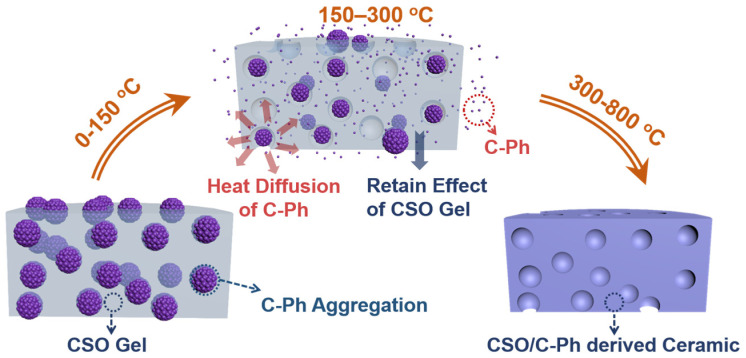
Schematic of the pyrolysis processes for fabricating SiOC ceramics.

The schematic in Figure 11 illustrates the pyrolysis processes of fabricated SiOC ceramics, using S10 as an example. Phase separation begins during CSO solidification via hydrosilylation, and the C-Ph phase is extruded from the CSO network and fixed in the resulting gel as a permanent morphology [19,20]. Subsequently, at pyrolysis temperatures of up to 150 °C, C-Ph volatilization and diffusion occurs. By diffusing through the gaps in the polymer chain, the C-Ph droplets embedded in the CSO gel gradually volatilize until they disappear by evaporation, while pores form at their original positions. During volatilization, some of the C-Ph is trapped by the CSO gel, whereas the rest diffuses to the outside. The captured portion participates in the ceramic processes and can be monitored based on the yield and carbon content of the ceramics. In summary, the phase separation between C-Ph and the CSO gel plays a critical role in the formation of the final macroporous SiOC ceramic.

**Figure 12:**
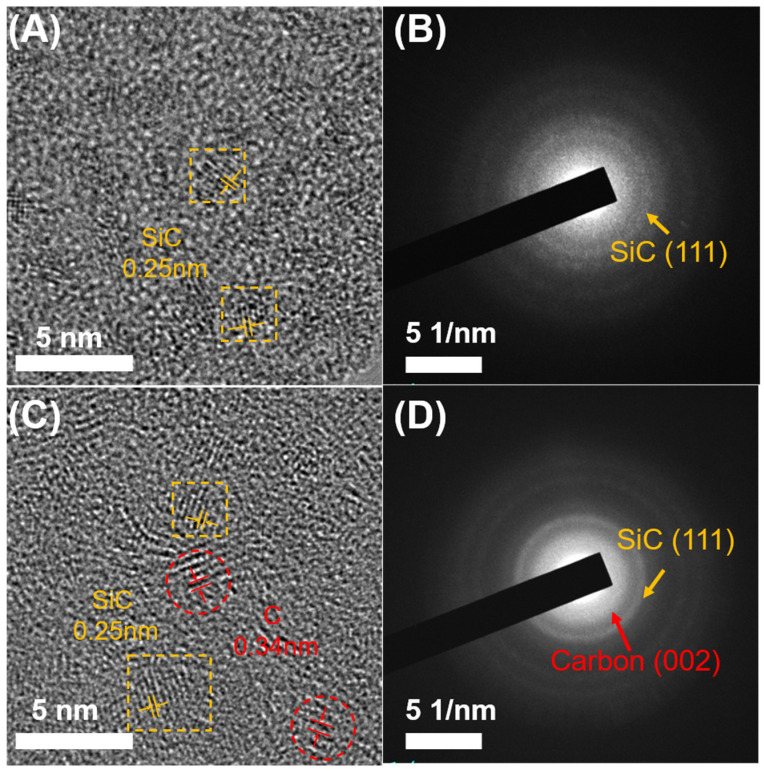
HRTEM and the corresponding SAED patterns of S0 (**A**,**B**) and S10 (**C**,**D**) pyrolyzed at 1400 °C.

To clearly confirm the distribution of C-Ph pyrolysis products in the ceramics, S0 (Figure 12A,B) and S10 (Figure 12C,D) were characterized using HRTEM and the corresponding SAED. S10 was selected because it has the highest carbon content, and S0 was used as the blank control. Moreover, the pyrolysis temperature of 1400 °C was selected because crystallization occurs at this temperature. The TEM images of both S0 and S10 show that many lattice structures were uniformly dispersed in the amorphous matrix. Abundant SiC and graphite crystals were observed in S10, whereas graphite crystals were not detected in S0. Moreover, the SAED pattern of S10 shows Debye rings corresponding to graphite crystals, which were not detected for S0. The corresponding XRD patterns are shown in Figure S5. The d values of the graphite crystals in S10 measured by SAED were consistent with those measured by XRD [29,30,31]. These results indicate that graphite crystals form after C-Ph pyrolysis and are uniformly dispersed in the ceramic matrix.

## 4. Conclusions

In summary, the molecular aggregation induced by C-Ph in precursor gels was used to fabricate macroporous SiOC ceramics. The initial C-Ph content in the precursor gels determined the final structure of the resulting gels and ceramics. The controllable formation of closed-pore and particle-packing structures were detected using SEM. Samples with particle-packing structures were characterized using a thermal constants analyzer, and a thermal conductivity of 27.4 mW m^−1^ K^−1^ was recorded. Moreover, some of the C-Ph was involved in pyrolysis, as demonstrated by TEM and carbon sulfur analysis, and the mechanism was investigated. This molecular aggregation strategy may develop a phenyl-substituted polysiloxane application for porous and thermal insulation materials.

## Figures and Tables

**Table 1:** Calculated yields of the CSO and residual C-Ph phases based on the yields of pure CSO (S0).

Sample	Ceramic Yield (wt%)	Calculated Yield of the CSO Phase (wt%) ^a^	Calculated Residual C-Ph Phase Content (wt%) ^b^
S0	83.2	83.2	0.0
S10	82.8	74.9	7.9
S30	68.5	58.2	10.3
S50	43.8	41.6	2.2
S70	28.3	25.0	3.3
S80	19.6	16.6	3.0

^a^ Yields of the CSO phase were calculated by multiplying the yields of pure CSO by the CSO content in the precursor gels. ^b^ Residual C-Ph phase contents were calculated by subtracting the calculated yields of the CSO phase from the ceramic yields.

**Table 2:** Properties of the fabricated ceramics.

Sample	Mercury Pressure Porosimetry		Bulk Density (g/mL)	Porosity (%) ^a^	Carbon Content (wt%)	Thermal Conductivity (mW m^−1^ K^−1^)
	Average Pore Diameter (nm)	Open Porosity (%)				
S0	17,065.5	8.1	1.78	20.2	25.4	130.7
S10	21,416.0	7.3	1.77	20.6	30.3	122.6
S30	8414.5	4.5	1.73	22.4	30.1	113.0
S50	---	---	---	---	28.4	---
S70	2337.9	47.4	0.83	62.8	29.3	39.8
S80	2657.6	54.7	0.71	68.2	28.7	27.4

^a^ The density of the completely densified SiOC ceramics was determined to be 2.23 g/cm^3^.

## Data Availability

Not applicable.

## Supplementary Materials

The following supporting information can be downloaded at: https://www.mdpi.com/article/10.3390/polym15122676/s1, Table S1: The summary of the fitted peak parameters of narrow XPS scan spectra (Si) of samples; Table S2: The values of area integral for Raman fitting curves of S70 pyrolysis at different temperatures. Figure S1: Magnification SEM images of the fractural cross sections of the precursor gels with different C-Ph contents. (A–C) represent 50, 70, and 80 wt%, respectively. Figure S2: SEM-EDX mapping of S70, (A) SEM image of S70 with corresponding EDX maps for: B (C, 29.4 wt%), C (O, 21.9 wt%), and D (Si, 48.7 wt%), respectively. The scale label in Figure S2A represents 20 μm. Figure S3: Magnification SEM images of the fractural cross sections of SiOC ceramics S0 (A), S10 (B), S30 (C), S50 (D), S70 (E), and S80 (F) pyrolyzed at 800 °C. Figure S4: (A–F) Magnification SEM images of the surfaces of S0 (A and B), S10 (C), S30 (D), S70 (E), and S80 (F). Figure S5: XRD patterns of S0 and S10 that pyrolysis at 1400 °C.
