# Supplementary material for: Molecular Aggregation Strategy for Pore Generation in SiOC Ceramics Induced by the Conjugation Force of Phenyl
Source: Polymers (Basel). 2023 Jun 14;15(12):2676. doi: 10.3390/polym15122676 (PMC10302902; doi:10.3390/polym15122676)
Supplement: Supplementary file 1 [file polymers-15-02676-s001.zip › polymers-2439173-supplementary.pdf]

*Article*

# Molecular Aggregation Strategy for Pore Generation in SiOC Ceramics Induced by the Conjugation Force of Phenyl

## S1. Results and Discussion

**Table S1.** The summary of the fitted peak parameters of narrow XPS scan spectra (Si) of samples.

| Sample | Chemical Bond | Position | FWHM | Area | Sample | Chemical Bond | Position | FWHM | Area |
|---|---|---|---|---|---|---|---|---|---|
| S-0 | Si-C | 101.23 | 1.40 | 23000.7 | S70 | Si-C | 101.40 | 1.50 | 16477.0 |
| | Si-O | 102.13 | 1.63 | 26959.7 | | Si-O | 102.32 | 1.86 | 24741.2 |
| | Si-C | 101.23 | 1.40 | 16228.2 | S80 | Si-C | 101.40 | 1.50 | 13747.5 |
| S-10 | Si-O | 102.13 | 1.63 | 20800.0 | | Si-O | 102.13 | 1.82 | 25141.8 |
| | Si-C | 101.23 | 1.40 | 17700.1 | None | | | | |
| S-30 | Si-O | 102.13 | 1.63 | 26059.3 | | | | | |

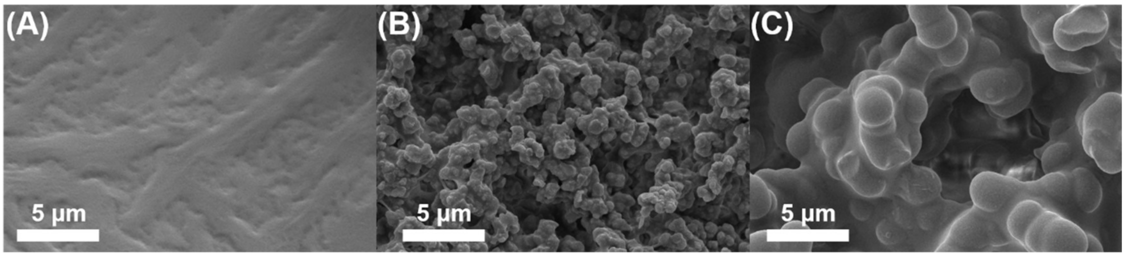


**Figure S1.** Magnification SEM images of the fractural cross sections of the precursor gels with different C-Ph contents. (A–C) represent 50, 70, and 80 wt%, respectively.

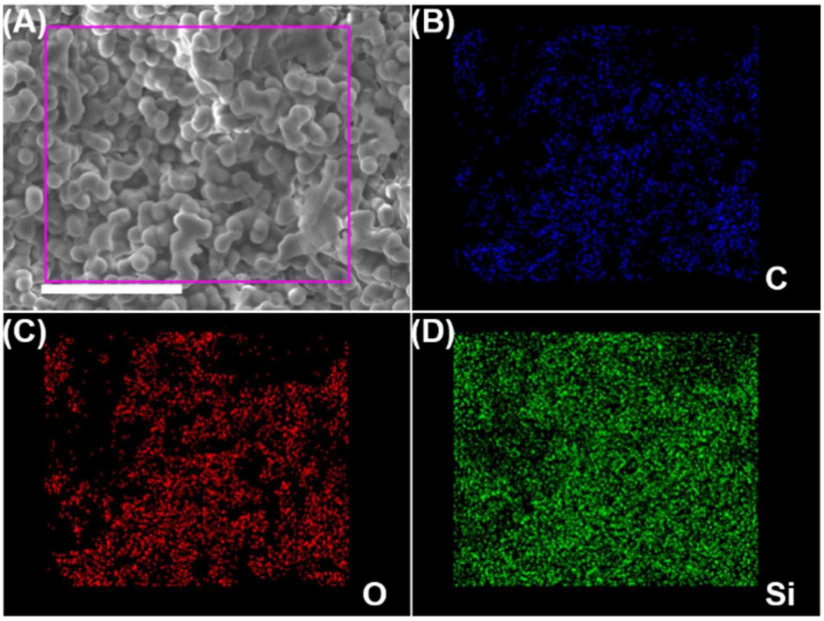


**Figure S2.** SEM-EDX mapping of S70, (A) SEM image of S70 with corresponding EDX maps for: B (C, 29.4 wt%), C (O, 21.9 wt%) and D (Si, 48.7 wt%), respectively. The scale label in Figure S2A represent 20 μm.

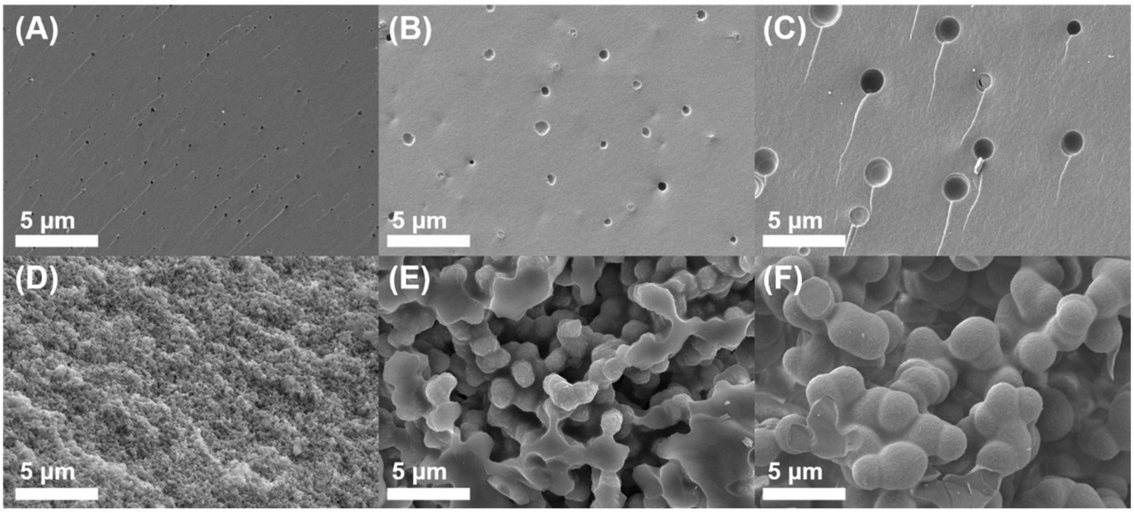


**Figure S3.** Magnification SEM images of the fractural cross sections of SiOC ceramics S0 (A), S10 (B), S30 (C), S50 (D), S70 (E), and S80 (F) pyrolyzed at 800 °C.

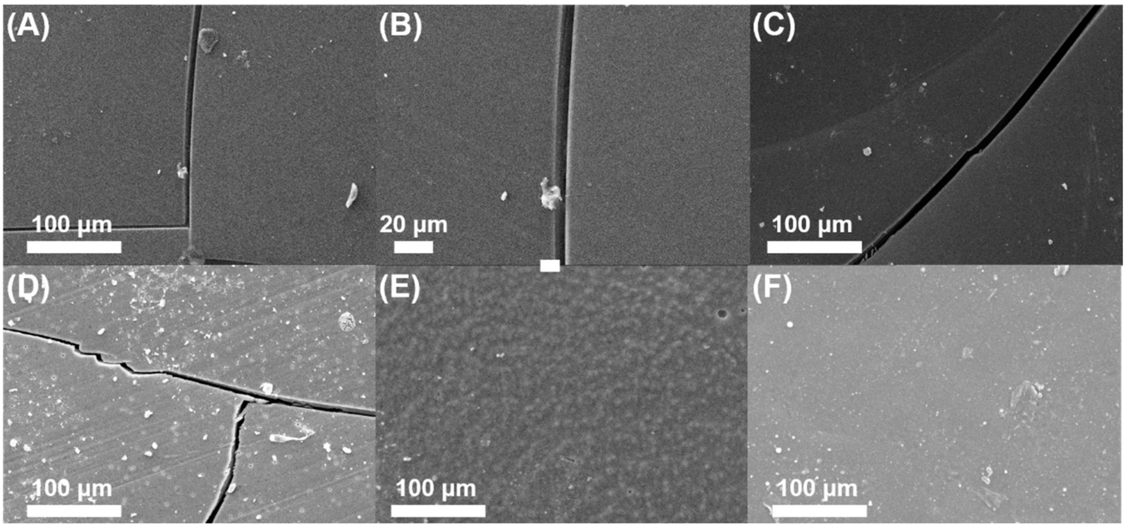


**Figure S4.** (A–F) Magnification SEM images of the surfaces of S0 (A and B), S10 (C), S30 (D), S70 (E), and S80 (F).

**Table S2.** The values of area integral for Raman fitting curves of S70 pyrolysis at different temperature.

| Temperature °C | D1 | D2 | a-C | G | $I_{D1}/I_G$ | $I_{D2}/I_G$ | $La^b$ nm |
|---|---|---|---|---|---|---|---|
| 800 | 1052840 | 914039 | 389512 | 1187508 | 0.89 | 0.77 | 15.29 |
| 1000 | 917514 | 366127 | 300423 | 709505 | 1.29 | 0.52 | 10.55 |
| 1200 | 1408075 | 276410 | 371373 | 547084 | 2.57 | 0.50 | 5.29 |
| 1400 | 1356785 | 224567 | 202995 | 436595 | 3.11 | 0.51 | 4.37 |

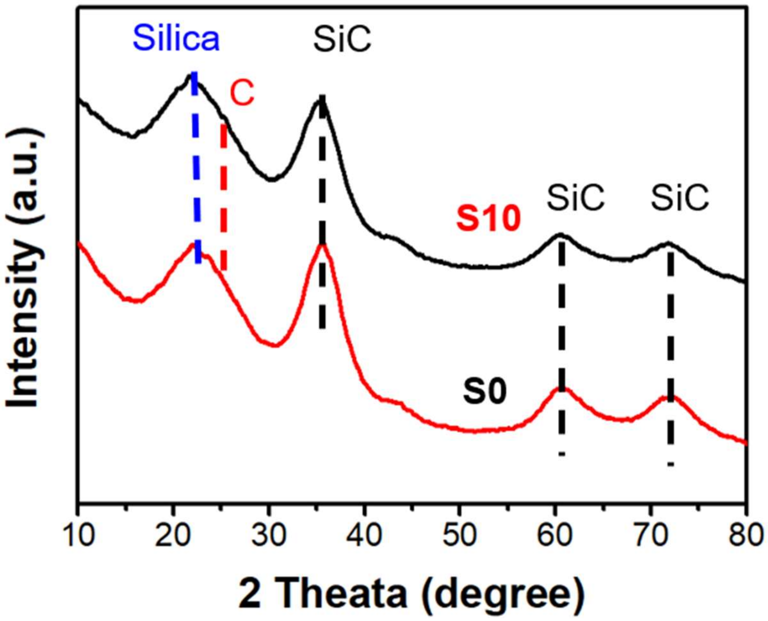


**Figure S5.** XRD patterns of S0 and S10 that pyrolysis at 1400 °C.
